# Deep learning-based Fast Volumetric Image Generation for Image-guided Proton FLASH Radiotherapy

**DOI:** 10.21203/rs.3.rs-3112632/v1

**Published:** 2023-07-26

**Authors:** Chih-Wei Chang, Yang Lei, Tonghe Wang, Sibo Tian, Justin Roper, Liyong Lin, Jeffrey Bradley, Tian Liu, Jun Zhou, Xiaofeng Yang

**Affiliations:** Emory University; Emory University; Memorial Sloan Kettering Cancer Center; Emory University; Emory University; Emory University; Emory University; Mount Sinai Medical Center; Emory University; Emory University

## Abstract

**Objective:**

FLASH radiotherapy leverages ultra-high dose-rate radiation to enhance the sparing of organs at risk without compromising tumor control probability. This may allow dose escalation, toxicity mitigation, or both. To prepare for the ultra-high dose-rate delivery, we aim to develop a deep learning (DL)-based image-guide framework to enable fast volumetric image reconstruction for accurate target localization for proton FLASH beam delivery.

**Approach:**

The proposed framework comprises four modules, including orthogonal kV x-ray projection acquisition, DL-based volumetric image generation, image quality analyses, and water equivalent thickness (WET) evaluation. We investigated volumetric image reconstruction using kV projection pairs with four different source angles. Thirty patients with lung targets were identified from an institutional database, each patient having a four-dimensional computed tomography (CT) dataset with ten respiratory phases. Leave-phase-out cross-validation was performed to investigate the DL model’s robustness for each patient.

**Main results:**

The proposed framework reconstructed patients’ volumetric anatomy, including tumors and organs at risk from orthogonal x-ray projections. Considering all evaluation metrics, the kV projections with source angles of 135° and 225° yielded the optimal volumetric images. The patient-averaged mean absolute error, peak signal-to-noise ratio, structural similarity index measure, and WET error were 75±22 HU, 19±3.7 dB, 0.938±0.044, and −1.3%±4.1%.

**Significance:**

The proposed framework has been demonstrated to reconstruct volumetric images with a high degree of accuracy using two orthogonal x-ray projections. The embedded WET module can be used to detect potential proton beam-specific patient anatomy variations. This framework can rapidly deliver volumetric images to potentially guide proton FLASH therapy treatment delivery systems.

## 1 Introduction

Proton therapy utilizes the physics characteristics of protons, which have well-defined ranges in a medium, to conformally deposit radiation energy to target volumes without exit doses^1,2^. This feature decreases the toxicity to healthy tissues such that patients who received proton therapy have lower risks of unplanned hospitalization compared to those who received photon treatment^3^. However, proton range uncertainty ^4^ requires additional margins for robust treatment planning^5,6^, which may compromise the sparing of healthy tissues when organs at risk (OAR) are closely adjacent to the treatment target. In the era of precision medicine, the critical question is how to minimize the radiation doses to OARs such that planning constraints are no longer dose-limiting for typical target prescription doses.

Favaudon *et al*. demonstrated that ultra-high dose-rate (≥ 40 Gy/sec) radiation, the so-called FLASH effect, (TCP) ^7^, can preferentially spare normal tissues from acute radiation-induced apoptosis, while maintaining similar tumor control ^8^. This promising finding can potentially make a paradigm shift in radiotherapy, and the FLASH effect has been explored in several settings since its discovery^9–11^. Proton FLASH therapy has been investigated regarding the feasibility of using the current commercial treatment delivery system and inverse planning^12–14^. Meanwhile, the efficiency and accuracy of image-guided systems are important due to high dose and ultra-high dose rate of FLASH delivery^15^. On-board fast imaging systems are essential to detect potential patient anatomy changes and for motion management, especially for patients with lung targets. However, the current proton on-board cone-beam computed tomography (CBCT) images require 30–60 seconds of scan time, and their quality can be compromised due to motion and artifacts.

Commercial proton machines, such as Varian ProBeam and IBA Proteus^®^ONE, include two kV x-ray sources with an image acquisition time of less than a second. The two orthogonal kV projections can potentially be acquired simultaneously, and the volumetric reconstruction method based on these projections will be free of motion and cavity artifacts. However, image reconstruction based on two projections is ill-conditioned^16^. This ill-posed problem poses a challenge for conventional image reconstruction methods. In contrast, deep learning (DL) has been demonstrated as a universal approximator^17^, and DL models feature in hierarchical learning to discover the underlying patterns behind the data^18^. A significant challenge of applying DL to medical volumetric image reconstruction is the identification of tumor regions due to information lost when superimposing three-dimensional (3D) volumetric images to 2D projections^19^.

Many researchers have investigated various DL models to reconstruct 3D volumetric images based on limited 2D information^20–22^. One approach uses deformable image registration techniques to register 2D and 3D images^23^. A recent development^24^ integrates DL and mechanical models to achieve real-time liver tumor localization. However, the previous literature is usually based on a single x-ray projection, and the robustness of DL models in terms of CT numbers for dose evaluation remains an open question.

This study proposes a DL-based image-guide framework to inform the potential proton FLASH treatment, including tumor position and patient anatomy changes. We use two orthogonal x-ray projections to provide additional information to enhance the predictability of DL models. Most importantly, we integrate a ray tracing-based water equivalent thickness (WET) evaluation module into the proposed framework for treatment feasibility investigation. This module can specifically detect potential anatomy changes corresponding to proton beams. To evaluate the proposed framework, we investigate under which conditions the volumetric images can be derived effectively, accurately, and robustly to support medical decision-making.

## 2 Materials and methods

### 2.1 Patient data

This work aims to develop an image-guided framework to manage patient anatomy and motion for accurate target localization before proton FLASH beam delivery. Since treatment of lung targets usually requires motion management, we identified 30 patients from an institutional database with 4D CT for the framework demonstration. Each 4D CT dataset includes ten respiratory phases (CT) acquired from a Siemens SOMATOM Definition AS scanner using a 120-kVp spectrum. The CT dataset for each phase has a resolution of 0.98×0.98×3.0 mm^3^. Synthetic kV x-ray projections were used to investigate the feasibility of the proposed framework, and the projections were generated based on Varian (Varian Medical Systems, Palo Alto) kV image system. The digital x-ray panel includes 768×1024 detector channels with a spacing of 0.39 mm. The synthetic kV projections were acquired from four different angle pairs, including 112°/202°, 135°/225°, 157°/247°, and 180°/270°, based on the x-ray source position.

### 2.2 Deep learning-based image-guided framework for proton FLASH treatment

**Figure 1:**
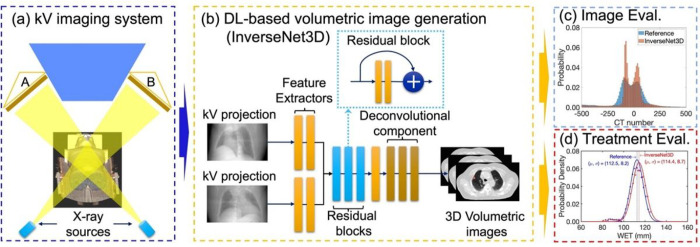
Deep learning-based image-guided framework for proton therapy FLASH treatment, consisting of four modules: (a) kV image system with two digital x-ray panels A and B to acquire two orthogonal projections simultaneously, (b) Deep learning (DL)-based volumetric image generation using orthogonal kV projections (InverseNet3D is implemented in this module), (c) image evaluation based on CT numbers to ensure the integrity of generated volumetric images without systematic shift of voxel intensity, and (d) treatment evaluation based on WET to detector potential anatomy changes.

**Figure 2:**
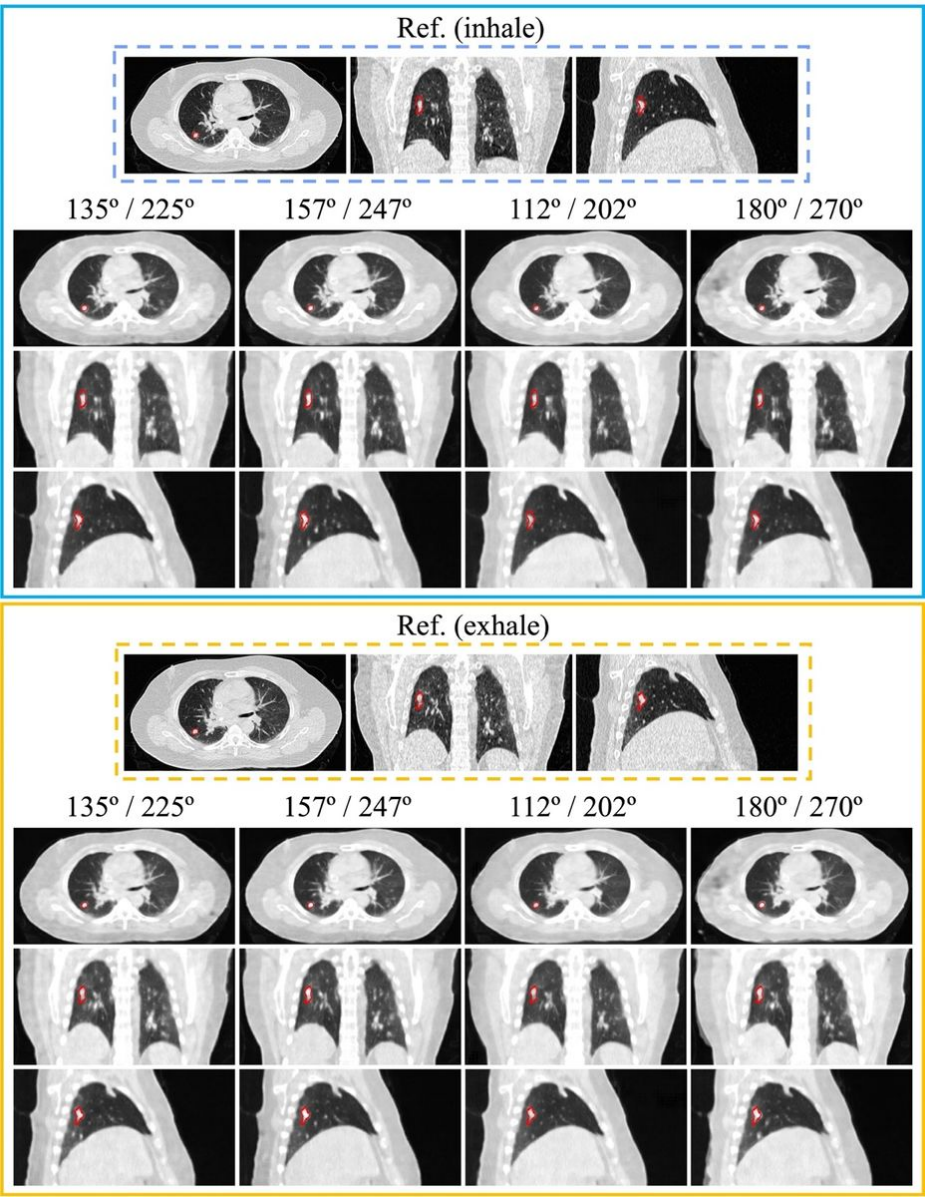
Reference (Ref.) and generated (InverseNet3D) volumetric images seen in transversal, coronal, and sagittal views for case 1 for both inhale and exhale phases using orthogonal kV projections at various source angle pairs. The window level of each image is [−1000, 200] Hounsfield units (HU). The lesion ROI is marked in red.

**Figure 3:**
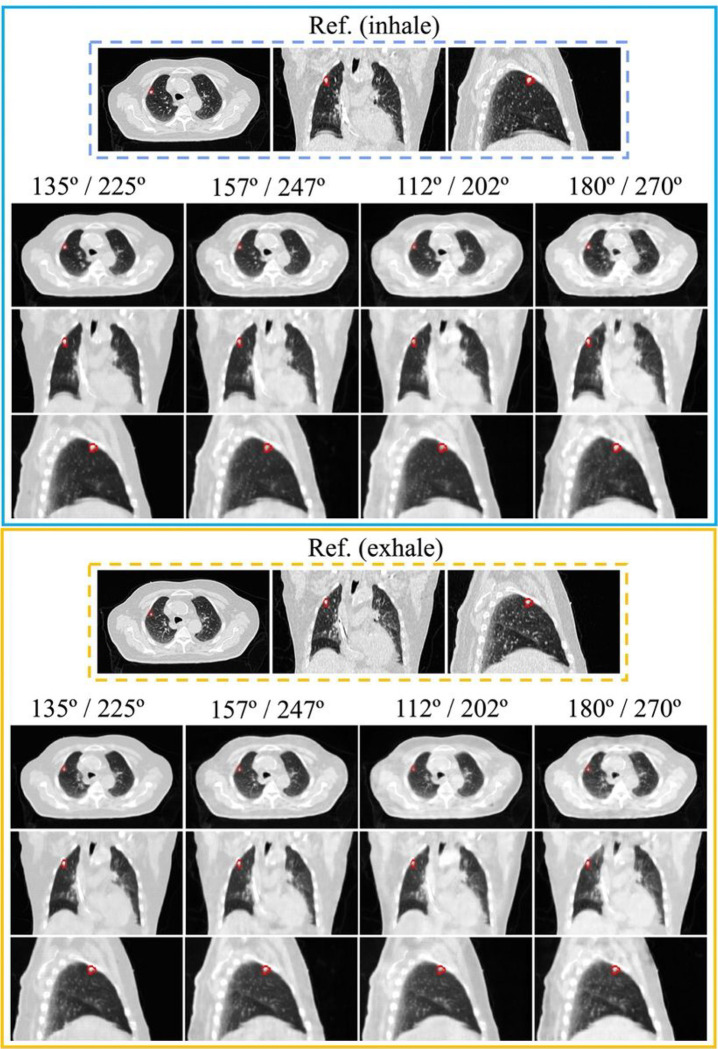
Reference and generated (InverseNet3D) volumetric images seen in transversal, coronal, and sagittal views of case 2 for inhale and exhale phases using orthogonal kV projections at various source angle pairs. The window level of each image is [−1000, 200] Hounsfield units (HU). The lesion ROI is marked in red.

**Figure 4:**
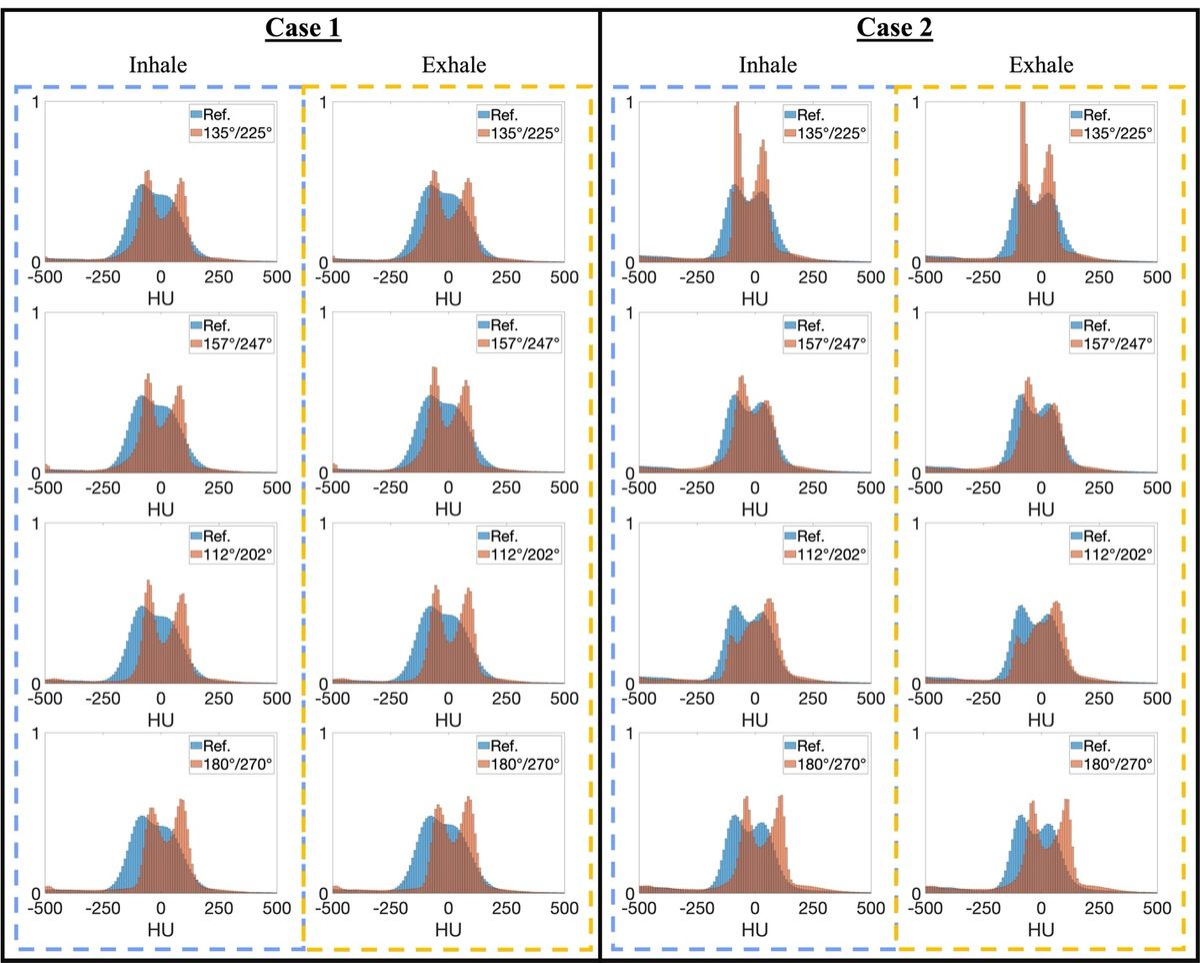
Histograms of CT number distributions for inhale and exhale phases from the reference and generated (InverseNet3D) volumetric images of case 1 and case 2 using orthogonal kV projections at various source angle pairs including 135°/225°, 157°/247°, 112°/202°, and 180°/270°.

The image-guided system is essential for proton FLASH treatment due to its ultra-high dose rate. Figure 1 depicts the DL-based image-guided framework for proton FLASH treatment, including four modules to ensure the accuracy of target dose delivery. Figure 1(a) shows the kV image system of a conventional proton treatment machine, including two digital x-ray panels acquiring orthogonal projections. Figure 2(a) displays the volumetric image generation module using two orthogonal x-ray projections. A deep learning model, InverseNet3D, was implemented to transform two orthogonal images into 3D images inversely. Section 2.2.1 gives the details of InverseNet3D regarding the model form and model parameters. Figure 3(c) shows the image evaluation module to quantify the integrity of generated volumetric images, conserving image features from reference CT such as CT number histograms, image structures, noise levels, and statistical parameters. Section 2.2.2 gives the details of each evaluation metric. Figure 4 depicts the module for treatment evaluation to detect potential patient anatomy changes. The assessment is based on WET comparisons between the reference CT and generated volumetric images, and the details of comparisons are described in Section 2.2.3. The framework performance has been evaluated regarding image quality and proton characteristics (i.e., WET). The validated framework is expected to deliver on-board volumetric images for localization and delivery of proton FLASH treatment.

#### 2.2.1 InverseNet3D

Figure 1(b) depicts the hierarchical structure of the InverseNet3D to transform two orthogonal x-ray projections inversely. InverseNet3D includes three components to infer volumetric images from 2D projections. Initially, the feature extractors, built by convolutional neural networks (CNN), are used to identify the local patterns from the x-ray projections. The second component includes multiple residual blocks to prevent gradient vanishing and to enhance the performance of model learning during the error backpropagation processes. Each residual block’s fundamental unit is composed of two convolutional layers with a single residual layer. Ultimately, the deconvolution component upscales the dimension of local feature images received from the residual blocks to conserve the dimensions of volumetric images (CT) from patients. The detailed model structures and parameters are given in Appendix A.

To investigate the model robustness, the InverseNet3D was trained using leave-phase-out patient-specific training with 4D CT datasets from 30 patients. Supervised loss functions were used to train the model, including mean absolute error (MAE) loss for voxel-wised image intensity learning and gradient loss for image edge learning. Tensorflow v1.15.0^25^ was used to implement model hierarchy, optimization, and data preprocessing. The simulation environment included an NVIDIA Tesla V100 GPU.

#### 2.2.2 Image evaluation

Three metrics were used to evaluate the quality of each volumetric image voxel generated by InverseNet3D. Eq. (1) gives the mean errors (ME) where the x,i,N,DL, and ref denote the voxel CT number, the i^th^ voxel, total voxels, generated images by InverseNet3D, and reference images, respectively. The CT number unit is in Hounsfield units (HU). ME can quantify systematic intensity shifts of the generated volumetric images from the reference. Eq. (2) defines the MAE to determine the global quality of the generated images. Eq. (3) gives the peak signal-to-noise ratio (PSNR) to evaluate the reconstruction quality of the InverseNet3D from orthogonal 2D projections. The structural similarity index measure (SSIM), as previously described ^26^, was used to quantify the structural consistency of lesion region between the reference and InverseNet3D. The histogram of CT numbers was also evaluated to investigate differences in global profiles. All evaluation metrics were implemented using MATLAB R2021a.

1 $$ME=\frac{1}{N}\sum_{i=1}^{N}\left(x_{i,DL}-x_{i,ref}\right)$$

2 $$MAE=\frac{1}{N}\sum_{i=1}^{N}\left|x_{i,DL}-x_{i,ref}\right|$$

3 $$PSNR=10log_{10}\left[\frac{{\text{max}}^{2}\left(x_{ref}\right)}{\frac{1}{N}\sum_{i=1}^{N}{\left(x_{i,DL}\;-\;x_{i,ref}\right)}^{2}}\right]$$

#### 2.2.3 Treatment evaluation

Due to the ultra-high dose rate of proton FLASH therapy, patient anatomy changes are critical and may significantly impact treatment quality. The WET^27–29^ can be used to quantify potential inter-fractional or intra-fractional anatomic changes during proton FLASH treatment. To maximally spare organs at risk, an anteroposterior beam is commonly used in the treatment^30^, and we explored the WET for this beam. We implemented a ray tracing-based WET algorithm^31,32^ to derive the WET within the target region using MATLAB R2021a. The essential CT-number-to-relative-stopping-power (RSP) conversion table^33,34^ is given in Appendix B for WET calculation.

The RSP can be derived from CT numbers using HLUT in Appendix B. Gaussian fitting was used to fit raw WET histograms to minimize the uncertainty due to image noises. The WET uncertainty can be caused by patient anatomy changes and inconsistent image acquisition modalities for treatment planning and daily image guidance. This work aims to investigate the WET uncertainty from an image-guided system (i.e., the quality of generated images from InverseNet3D). Eq. (4) defines the difference of WET $(\left.\Delta WET\right)$ within a given region of interest (ROI) where i,N,DL,ref denote the i^th^ voxel in the ROI, total voxels in the ROI, InverseNet3D generated images, and reference images. Eq. (5) defines the relative difference of WET $(\left.\varepsilon_{WET}\right)$ within a given ROI. We focused on the target ROI in this work since the target volume is directly associated with proton beams. In order to increase tumor control probability, we need to ensure accurate dose delivery to the target volume.

4 $$\text{Δ}WET=\frac{1}{N}\sum_{i=1}^{N}\left(WET_{i,DL}-WET_{i,ref}\right)$$

5 $$\epsilon_{WET}=\frac{\text{Δ}WET}{\frac{1}{N}\sum_{i=1}^{N}WET_{i,ref}}\times 100\text{\%}$$

## 3 Results

### 3.1 Volumetric image generation using orthogonal kV projections from different angles

We investigated the framework performance using 4D CT images from 30 patients with 4 orthogonal projection pairs including 180°/270°, 135°/225°, 157°/247°, and 112°/202°. The framework synthesized CT images were evaluated using image- and proton-related metrics. Table 1 shows the results of all patients based on the projections from the source angle pair of 135°/225°, and the results indicate that each metric’s standard deviations (SD) are usually slight between respiratory phases. The SD values are generally smaller than 5% of the mean values. The framework evaluation using more source angle pairs are given in Table C1-C3 in Appendix C. We systematically analyzed the phase-averaged outcomes of CT image synthesis for each patient using 2D orthogonal projections generated from 4 different source angle pairs.

Table 2 shows the patient average evaluation results of generated image quality using InversereNet3D with four orthogonal projection pairs. The 180°/270° projection pair results in the minimum ME, which is approximate − 0.3% (−3.3/1000×100%) error regarding material properties because of *CT number (HU) = 1000(μ−1),* where *μ* is the relative linear attenuation coefficient material to water. The percentage ME values for the other three orthogonal projection pairs are − 1.6%, −0.9%, and 0.4%, corresponding to the angle pairs of 135°/225°, 157°/247°, and 112°/202°.

The generated volumetric image from the 112°/202° projection results in the optimal MAE, while the 180°/270° projection causes the maximum MAE. The PSNR analyses show that both 157°/247° and 180°/270° projections have comparable 3D reconstruction image quality. The volumetric image inferred from the 112°/202° projection causes the minimal PSNR. The minimum SSIM was achieved by the image generated by using InverseNet3D with the 157°/247° projections. The volumetric images inferred from the 180°/270° projection results in the worst SSIM value.

To demonstrate the visual results of the proposed framework using all orthogonal projection pairs, we formulated case 1 and case 2 using the synthetic image sets from patients 3 and patient 15. The selection was based on 5% (patient 3) and 95% (patient 15) percentile of PSNR results from all patients. Figure 2 demonstrates that InverseNet3D can generate volumetric images from four orthogonal projection pairs; case 1 is seen in transversal, coronal, and sagittal views. The lesion locations are marked in red for both reference and generated images. Volumetric images derived from all orthogonal projection pairs were used to reconstruct the patient’s anatomy and identify the tumor in the lung. The heart and liver can be clearly identified from the transversal and sagittal images. Figure 3 depicts the reference and generated images for case 2, which has a smaller body and tumor size than the patient shown in Fig. 2. The lesion target and important organs can be recognized from the volumetric images, such as the tumor, heart, aorta, vena cava, and spine.

Figure 4 shows the histogram comparisons of CT numbers between the reference and InverseNet3D-generated images for cases 1 and 2. Figure 4 shows that the generated images from the 135°/225° projection pair align well with the reference histograms for both inhale and exhale scenarios. The 157°/247° and 112°/202° projection pairs exhibit a slight shift from the reference histogram. Compared to other projection pairs, apparent histogram shifts of generated images from the 180°/270° projection pair can be observed in Fig. 4.

### 3.2 Treatment evaluation using water equivalent thickness (WET)

The phase-averaged WET analysis results for all 30 patients with 4 projection angle pairs are given in Table 1 and Table C1-C3 in Appendix C. Table 3 summarizes the patient-averaged WET difference (ΔWET) and relative WET difference (εWET) results calculated by Eq. (4) and Eq. (5) for generated volumetric images by InverseNet3D using multiple orthogonal projection pairs. The volumetric images from the 180°/270° projection pair result in a minimal ΔWET of −0.7 mm. However, its standard deviation is approximately twice that of images generated from the 135°/225° projection pair. The images generated from the 135°/225° projection pair also lead to the minimal standard deviations of ΔWET and εWET with values of 3.7 mm and 4.1%.

**Figure 5:**
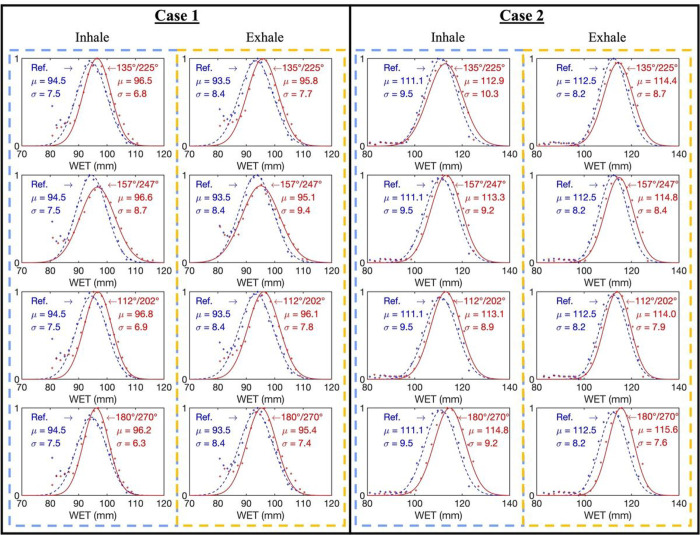
Histograms of WET distributions for inhale and exhale phases from the reference (Ref.) and generated (InverseNet3D) volumetric images of case 1 and case 2 using orthogonal kV projections at various source angle pairs of 135°/225°, 157°/247°, 112°/202°, and 180°/270°.

Figure 5 depict the WET distributions within the target ROI for case 1 and case 2 at inhale and exhale phases using InverseNet3D images with different orthogonal projection pairs. The absolute mean WET differences between inhale and exhale phases are 1 mm and 1.4 mm for case 1 and case 2. For case 1, no significant variation was found for mean WET values. However, Fig. 5 shows that the images generated from the 157°/247° projection pair yield the maximum standard deviation of WET. The standard deviation is approximately 2 mm more extensive than the images derived from other projection pairs. For case 2, Fig. 5 displays that the images generated from 180°/270° projection pair cause an approximate mean WET shift of 1.5 mm, compared to the 135°/225° projection pair images.

## 4 Discussion

Proton FLASH treatment can potentially create a paradigm shift in radiotherapy due to its capability to deliver ultra-high dose-rate irradiation while maximally reducing the toxicity for normal tissues. The inherent ultra-high dose rate feature makes the proton FLASH favorable to stereotactic body radiation therapy, which can help proton therapy become affordable and benefit patients by avoiding short-term and long-term side effects. As the decrease of fractionated treatment times, the prescription dose per fraction will inevitability increase such that an accurate image-guided system becomes essential. This work demonstrates a DL-based image-guided framework to generate fast volumetric images without motion artifacts due to the use of two instant-captured orthogonal x-ray projections. Acquiring kV x-ray projections is less than a second, and then the proposed framework can almost instantaneously deliver volumetric images for treatment evaluation.

In contrast, the current proton on-board CBCT^35^ requires approximately 35 and 60 seconds for full-fan and half-fan scans. InverseNet3D is currently implemented in the framework. Based on the retrospective patient study, all 30 patients’ anatomy can be identified from the generated volumetric images, including tumor tissues. The proposed framework delivers patient-averaged MAE of approximately 75 HU, which improves the image quality by at least 20% from the previous work^36,37^. Most importantly, the framework integrates a WET analysis module for the treatment evaluation, and a patient-averaged ΔWET of ~ 1 mm can be achieved in the present work. The analyses of WET not only indicate the image quality but also monitor the potential anatomy changes on the treatment beam path, which potentially increases the usability of the proposed framework to inform proton FLASH treatment.

Table 1 and Table C1-C3 provides the phase-averaged evaluation for the generated volumetric images from each patient using a leave-phase-out cross-validation method to investigate the robustness of the InverseNet3D module in the framework. Since each patient included ten raspatory phases, ten variants of InverseNet3D were trained for each patient, and a total of 300 variants of InverseNet3D were explored for a 30-patient cohort to ensure the method’s robustness. Table 1 and Table C1-C3 indicates that the intra-patient standard deviations of each evaluation metric are usually smaller than 5% of their mean values. This result shows the proposed framework can consistently infer volumetric images from orthogonal x-ray projections. Table <link rid=“tb2”>2</link>−2 provide the patient-averaged image and WET evaluation to investigate the inter-patient variability. Table 2 shows that the 180°/270° projection pair yield the most considerable inter-patient image intensity variation due to the largest MAE of 80 ± 24 HU. The 180°/270° projection pair also results in the smallest SSIM, while the other three projection pairs have comparable SSIM values. Table 3 also shows that the 180°/270° projection pair makes the largest εWET standard deviation of 6.8%. Meanwhiles, Table 3 indicates 135°/225° projection pair can derive the volumetric images with minimal inter-patient variation when considering the uncertainty. The 157°/247° and 112°/202° projection pairs show comparable results. Conclusively, these findings suggest avoiding lateral x-ray projections for image inference. The lateral projections may be less informative because the projection area is smaller compared to other projection areas from different source angles.

Figure 2–3 demonstrate the quality of volumetric image reconstruction for patients with different body sizes, target volumes, and lesion locations. The generated 3D images from the framework can successfully conserve the patient’s anatomy, and both tumors and organs at risk can be identified. The results indicate that it is feasible to use the proposed method to localize treatment regions and guide the delivery of proton beams for potential proton FLASH therapy. Another potential volumetric image application is for CT artifact reduction induced by surgical implants^38–40^. The kV x-ray projection provides better image quality compared to CT images when metal implants are present in patients. Reconstructing the volumetric images from kV projections can potentially mitigate the impact of photon starvation, which can cause metal artifacts on CT images. High-quality volumetric images can also increase the accuracy of material characterization to make an accurate dose evaluation^41^ and support medical decision-making.

Inverse model inference from data (DL) is ill-posed, and the solution cannot satisfy the Hadamard principle of well-posedness^42^. Cross-validation is essential to ensure a robust DL model^43^. However, this approach requires a considerable amount of numerical experiment time. Future investigation will likely focus on developing advanced validation experiments using human-mimicking phantoms and state-of-the-art instrumentation ^44^ to quantify proton range uncertainty. Then the experiment data can be used to identify which DL models can work compatibly, effectively, and robustly with the proposed image-guided framework for proton FLASH radiotherapy.

## 5 Conclusions

A DL-based image-guided framework has been demonstrated for generating volumetric images using two orthogonal kV x-ray projections. The approach includes image quality and WET analyses for potential online dose evaluation and potential inter-fractional and intra-fractional anatomy changes. The proposed framework can inherently avoid motion artifacts and deliver instantaneous patient anatomy and target position to inform potential proton FLASH treatment.

## Figures and Tables

**Table 1:** Evaluation metrics of volumetric image quality generated from InverseNet3D using orthogonal images with the source angle pair of 135°–225°. The metrics include ME, MAE, PSNR, and SSIM. All metrics are averaged over all phases for each patient. ME and MAE are evaluated for the whole volume, and PSNR and SSIM are computed for the target contour. The difference and relative difference of water equivalent thicknesses (ΔWET/ε_WET_) are calculated within the target contour for an anteroposterior proton beam.

Patient	ME (HU)	MAE (HU)	PSNR (dB)	SSIM	ΔWET (mm)	ε_WET_
1	−8.5 ± 0.2	42.3 ± 0.8	22.3 ± 0.6	0.967 ± 0.011	−2.6 ± 0.2	−3.7 ± 0.2%
2	11.6 ± 0.5	50.2 ± 0.6	20.5 ± 0.5	0.978 ± 0.002	−2.8 ± 0.2	−2.0 ± 0.2%
3	34.2 ± 0.4	59.3 ± 0.3	14.7 ± 0.2	0.979 ± 0.005	1.3 ± 0.2	1.8 ± 0.3%
4	−37.8 ± 2.9	50.3 ± 3.7	12.2 ± 0.7	0.961 ± 0.012	−2.2 ± 0.2	−4.4 ± 0.4%
5	0.5 ± 0.3	53.0 ± 0.1	13.7 ± 0.1	0.989 ± 0.003	4.1 ± 0.1	5.2 ± 0.1%
6	−23.7 ± 0.1	45.2 ± 0.2	21.1 ± 0.3	0.960 ± 0.007	−2.2 ± 0.1	−5.6 ± 0.2%
7	−13.4 ± 0.7	76.9 ± 3.1	17.2 ± 0.5	0.967 ± 0.010	−1.2 ± 0.3	−3.1 ± 0.7%
8	−22.3 ± 1.1	96.1 ± 4.1	16.9 ± 0.4	0.944 ± 0.009	−2.3 ± 0.7	−2.6 ± 0.8%
9	−5.7 ± 0.3	88.5 ± 7.1	14.9 ± 0.4	0.892 ± 0.016	2.7 ± 0.9	2.2 ± 0.8%
10	−4.2 ± 1.0	99.1 ± 0.6	19.0 ± 0.2	0.901 ± 0.004	−7.1 ± 0.8	−7.4 ± 0.5%
11	−11.0 ± 0.5	102.8 ± 1.3	15.8 ± 0.1	0.866 ± 0.005	0.1 ± 0.3	0.1 ± 0.3%
12	−3.0 ± 0.4	84.7 ± 2.2	15.4 ± 0.4	0.900 ± 0.006	−5.8 ± 0.4	−7.6 ± 0.5%
13	−4.7 ± 0.6	69.9 ± 9.3	17.7 ± 1.4	0.988 ± 0.007	1.3 ± 1.0	4.5 ± 3.5%
14	−5.8 ± 0.5	84.0 ± 0.4	18.6 ± 0.1	0.882 ± 0.007	0.7 ± 0.3	2.1 ± 0.9%
15	−5.3 ± 0.3	38.5 ± 0.1	22.3 ± 0.2	0.979 ± 0.005	1.3 ± 0.3	1.3 ± 0.3%
16	−73.0 ± 0.4	94.5 ± 0.9	19.0 ± 0.2	0.992 ± 0.009	−0.9 ± 0.2	−3.3 ± 0.5%
17	14.4 ± 0.3	55.7 ± 0.7	21.1 ± 0.5	0.899 ± 0.004	−4.8 ± 0.5	−5.6 ± 0.5%
18	−42.8 ± 4.2	57.0 ± 5.8	18.8 ± 0.4	0.923 ± 0.005	−3.1 ± 1.0	−2.8 ± 0.9%
19	−6.1 ± 0.3	47.1 ± 0.8	21.0 ± 0.4	0.951 ± 0.003	−2.5 ± 0.1	−5.6 ± 0.1%
20	−21.0 ± 0.4	48.5 ± 0.3	32.8 ± 0.1	0.991 ± 0.002	−0.4 ± 0.1	−0.7 ± 0.1%
21	−28.0 ± 0.8	71.2 ± 0.8	21.4 ± 0.3	0.939 ± 0.012	−1.5 ± 1.5	−2.9 ± 2.8%
22	−30.0 ± 0.9	100.9 ± 1.0	19.2 ± 0.2	0.993 ± 0.001	0.1 ± 0.1	0.3 ± 0.3%
23	−17.7 ± 0.8	80.0 ± 0.9	20.1 ± 0.1	0.979 ± 0.001	−11.6 ± 1.3	−7.0 ± 0.7%
24	−22.0 ± 1.2	96.0 ± 1.5	21.4 ± 0.1	0.911 ± 0.010	1.1 ± 0.7	1.0 ± 0.6%
25	−11.9 ± 0.3	76.0 ± 1.3	15.4 ± 0.1	0.828 ± 0.074	5.1 ± 0.7	4.4 ± 0.6%
26	−29.4 ± 0.6	103.8 ± 0.7	18.6 ± 0.2	0.901 ± 0.009	−1.7 ± 1.1	−2.2 ± 1.4%
27	−22.3 ± 1.3	87.5 ± 3.1	20.0 ± 0.7	0.945 ± 0.004	−1.0 ± 0.7	−2.4 ± 1.6%
28	−32.4 ± 0.5	103.8 ± 0.3	21.6 ± 0.04	0.945 ± 0.005	2.1 ± 0.4	5.3 ± 1.0%
29	−20.0 ± 0.4	105.5 ± 1.9	18.4 ± 0.3	0.892 ± 0.004	3.5 ± 0.9	7.3 ± 1.9%
30	−23.9 ± 1.4	100.0 ± 3.9	18.7 ± 0.6	0.898 ± 0.017	−9.2 ± 2.0	−4.8 ± 1.0%

**Table 2:** Evaluation metrics of volumetric image quality generated from InverseNet3D using mean error (ME), mean absolute error (MAE), peak signal-to-noise ratio (PSNR), and structural similarity index measure (SSlM). All metrics are averaged over all patients given in Table 1 and Table C1-C3. ME and MAE are evaluated for the whole volume, and PSNR and SSIM are computed for the target contour.

	Orthogonal projections			
	135° / 225°	157° / 247°	112° / 202°	180° / 270°
ME (HU)	−15.5 ± 19.4	−9.2 ± 15.3	3.9 ± 17.8	−3.3 ± 15.0
MAE (HU)	75.6 ± 22.4	74.1 ± 21.3	73.4 ± 19.2	80.3 ± 24.0
PSNR (dB)	19.0 ± 3.7	20.2 ± 4.3	15.9 ± 3.6	20.4 ± 3.0
SSIM	0.938 ± 0.044	0.945 ± 0.046	0.941 ± 0.045	0.928 ± 0.058

**Table 3:** Treatment evaluation metrics of volumetric image quality generated from InverseNet3D using the difference and relative difference of water equivalent thicknesses (ΔWET/ε_WET_). All metrics are averaged over all patients given in Table 1 and Table C1-C3. The ΔWET and ε_WET_ are calculated within the target contour for an anteroposterior proton beam.

	Orthogonal projections			
	135° / 225°	157° / 247°	112° / 202°	180° / 270°
ΔWET (mm)	−1.3 ± 3.7	−1.6 ± 6.6	−0.8 ± 6.3	−0.7 ± 6.4
ε_WET_ (%)	−1.3 ± 4.1	−1.1 ± 5.3	0.3 ± 5.5	0.4 ± 6.8

## Data Availability

The datasets used and/or analysed during the current study available from the corresponding author on reasonable request.
